# Effects of Calcium Phosphate Nanocrystals on Osseointegration of Titanium Implant in Irradiated Bone

**DOI:** 10.1155/2015/783894

**Published:** 2015-01-22

**Authors:** Jun Yuan Li, Edmond Ho Nang Pow, Li Wu Zheng, Li Ma, Dora Lai Wan Kwong, Lim Kwong Cheung

**Affiliations:** ^1^Oral Rehabilitation, Faculty of Dentistry, The University of Hong Kong, Hong Kong; ^2^Oral Diagnosis and Polyclinics, Faculty of Dentistry, The University of Hong Kong, Hong Kong; ^3^Department of Clinical Oncology, Li Ka Shing Faculty of Medicine, The University of Hong Kong, Hong Kong; ^4^Oral and Maxillofacial Surgery, Faculty of Dentistry, The University of Hong Kong, Hong Kong

## Abstract

Radiotherapy may compromise the integration of implant and cause implant loss. Implant surface modifications have the possibility of promoting cell attachment, cell growth, and bone formation which ultimately enhance the osseointegration process. The present study aimed to investigate the effects of calcium phosphate nanocrystals on implant osseointegration in irradiated bone. Sixteen rabbits were randomly assigned into control and nano-CaP groups, receiving implants with dual acid-etched surface or dual acid-etched surface discretely deposited of nanoscale calcium-phosphate crystals, respectively. The left leg of all the rabbits received 15 Gy radiation, followed by implants placement one week after. Four animals in each group were sacrificed after 4 and 12 weeks, respectively. Implant stability quotient (ISQ), ratio of bone volume to total volume (BV/TV), bone growth rate, and bone-to-implant contact (BIC) were evaluated. The nano-CaP group showed significantly higher ISQ (week 12, *P* = 0.031) and bone growth rate (week 6, *P* = 0.021; week 9, *P* = 0.001) than that in control group. No significant differences in BV/TV and BIC were found between two groups. Titanium implant surface modified with CaP nanocrystals provides a potential alternative to improve bone healing around implant in irradiated bone.

## 1. Introduction

The success of implant osseointegration depends on the quality and quantity of the surrounding bone [1]. Radiotherapy has been considered as one of the predominant factors causing implant loss [2, 3]. It alters the circulation and metabolism of bone. Irradiation injures the small blood vessels leading to persistent hypoxia and reduces the quantity and activity of osteoblasts [4]. A number of studies showed that the failure rate of implants placed in irradiated bone was higher than those in nonirradiated bone [5–7]. This finding was confirmed in our previous study on a rabbit model [8]. The radiation at 15 Gy demonstrated a significantly adverse effect on implant stability and BV/TV.

Implant surface modifications may promote cell attachment, cell growth, and bone formation which ultimately enhances the osseointegration process. The surface modification includes physical method, chemical method, or a combination of both [9]. The CaP coated implant has demonstrated enhanced osteoconductive properties in normal bone [10, 11]. However, to our best knowledge no studies have investigated the osseointegration of CaP coated implant in irradiated bone.

The present study investigated the stability and osseointegration of CaP coated implant using our radiation compromised rabbit model [8].

## 2. Materials and Methods

### 2.1. Animal Care and Grouping

The animal experiment was approved by the Committee on the Use of Live Animals for Teaching and Research, The University of Hong Kong. Sixteen adult, male New Zealand white rabbits (8-9 months old) were randomly assigned into control and nano-CaP groups, eight in each. Rabbits in control group received implants with dual acid-etched surface (Osseotite, Biomet 3i Implant Innovations Inc., Palm Beach Gardens, FL, USA), while rabbits in nano-CaP group received implants with dual acid-etched surface discretely deposited of nanoscale calcium-phosphate crystals (Nanotite, Biomet 3i Implant Innovations Inc., Palm Beach Gardens, FL, USA). The timeline of treatment was presented in Table 1.

### 2.2. Radiation

Radiation on rabbits was performed by radiotherapists in Department of Clinical Oncology, Queen Mary Hospital, The University of Hong Kong, using the protocol reported in our previous study [8]. The tibial and femoral metaphysis region of left hind leg was subjected to a single dose of 15 Gy irradiation, whereas the other parts of the animals were protected. Electron beams of 9 MeV from a Varian Clinac 2100CD were delivered with a 15 × 15 cm^2^ applicator at a source to surface distance of 60 cm.

### 2.3. Implant Surgery

Implant surgery was performed one week after radiation. Under general anesthesia, parallel walled titanium implants with screw threads (3.25 mm × 8 mm) were placed in tibial and femoral metaphysic following the standardized protocol reported in our previous study [8]. Each animal received two implants on irradiated leg, one implant on tibia and one on femur. Totally 16 control implants and 16 nano-CaP implants were placed by the same surgeon. After surgery, appropriate antibiotics and analgesics were administered. Four rabbits in each group were sacrificed 4 weeks and 8 weeks after implant surgery, respectively. The implants together with 3–5 mm surrounding bone were harvested en bloc and fixed in 10% neutral formaldehyde. The timetable of radiation, implant surgery, fluorochrome labeling injection, and sacrifice is shown in Table 1.

### 2.4. Implant Stability Measurement

Resonance frequency analysis (RFA) device (Osstell; Integration Diagnostics, Savedalen, Sweden) was used to measure implant stability quotient (ISQ). Primary stability (ISQps) represented the ISQ value that was immediately measured after implant placement while secondary stability (ISQss) represented the ISQ value that was measured before sample retrieval.

### 2.5. Microcomputed Tomography (Micro-CT)

After being fixed in the formaldehyde for 2 days, the samples were wrapped in Parafilm (SERVA Electrophoresis GmbH, Heidelberg, Germany) and subjected to micro-CT assessment (Skyscan-1076 X-ray microtomograph, Skyscans, Kontich, Belgium). The samples were scanned at energy of 101 kV and intensity of 96 mA with a resolution of 9 mm pixel using an aluminum filter (1 mm). A threshold was selected to differentiate the titanium implant, bone, and background using the protocol described in our previous study [8]. The bone surrounding the implant at a distance of 180 *μ*m from the implant surface was analyzed, and the bone volume/total volume (BV/TV) was measured.

### 2.6. Fluorochrome Labeling

Three kinds of fluorochrome labeling, including alizarin red (25 mg/kg), calcin green (30 mg/kg), and oxytetracycline (50 mg/kg), were injected in chronological order (Table 1). For the rabbits sacrificed at week 4, the fluorochrome labeling was injected at week 1, week 2, and week 3, respectively. For the rabbits sacrificed at week 12, the fluorochrome labeling was injected at week 3, week 6, and week 9, respectively. After sacrifice, samples were embedded with methyl methacrylate (MMA, Technovit 7500, Kulzer, Hamburg, Germany). The embedded sample was sawed along the long axis of implant into a section with 200–500 *μ*m thick, which was then polished to about 100 *μ*m. The prepared slides were examined under fluorescent microscopy (FluoView FV 1000; Olympus, Tokyo, Japan). The bone growth rate was calculated as the average distance between every two fluorochrome-labeled lines over the known time interval of two corresponding injections.

### 2.7. Histomorphometric Analysis

After fluorescent microscopy examination, the slides were stained with toluidine blue for 30 min. Histomorphometrical analysis was performed using a camera-equipped light microscope system (Eclipse LV100POL, Nikon, Japan) and a computerized image analyzer (NIS-Elements AR 3.00). Bone-to-implant contact (BIC) was calculated as the length of the bone in direct contact with the implant over the implant length.

### 2.8. Statistical Analysis

All measurements were conducted by one trained, blinded, and calibrated examiner (single measures intraclass correlation coefficient >0.60). Repeated measures ANOVA (SPSS Inc., Chicago, IL, USA) were used to compare ISQ, BV/TV, bone growth rate, and BIC. Time and implant surface were defined as the factors. The level of significant difference was set at *P* ≤ 0.05.

## 3. Results 

### 3.1. Clinical Assessment

All sixteen rabbits completed the experiment uneventfully. No postoperative complications were observed till sacrifice. The implants remained submerged and soft tissues were clinically healthy.

### 3.2. Implant Stability

No significant difference in primary stability (ISQps) was found among all groups at baseline (Table 2). The secondary stability (ISQss) was significantly higher than ISQps in all the groups (*P* < 0.001). Significant difference of the secondary ISQ (ISQss) between control and nano-CaP groups was not detected at week 4 (*P* = 0.602), but at week 12 (*P* = 0.031). When compared groups from the two time points, the nano-CaP implant groups showed that ISQss at week 12 was significantly higher than that at week 4 (*P* = 0.004).

### 3.3. Micro-CT

**Figure 1:**
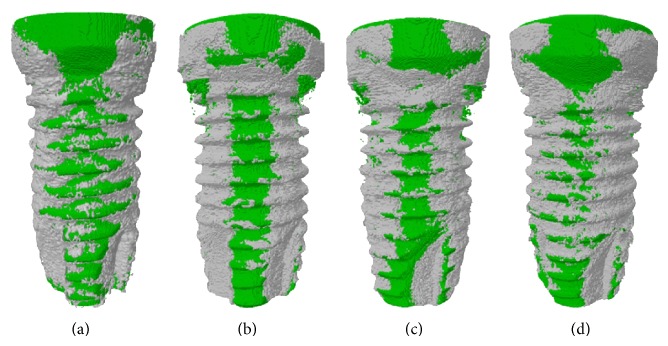
Micro-CT 3D images. (a) Control implant at week 4; (b) nano-CaP implant at week 4; (c) control implant at week 12; (d) nano-CaP implant at week 12. Green color represents implant surface and grey color represents bone.

The representative images of micro-CT three-dimensional (3D) models of bone formation around implants are shown in Figure 1. The BV/TV at week 12 was significantly higher than that at week 4 in both groups (control: *P* = 0.042; nano-CaP: *P* = 0.005) (Table 2). No significant difference of BV/TV was found between control and nano-CaP groups at week 4 (*P* = 0.579) and week 12 (*P* = 0.724).

### 3.4. Fluorescence Observation

**Figure 2:**
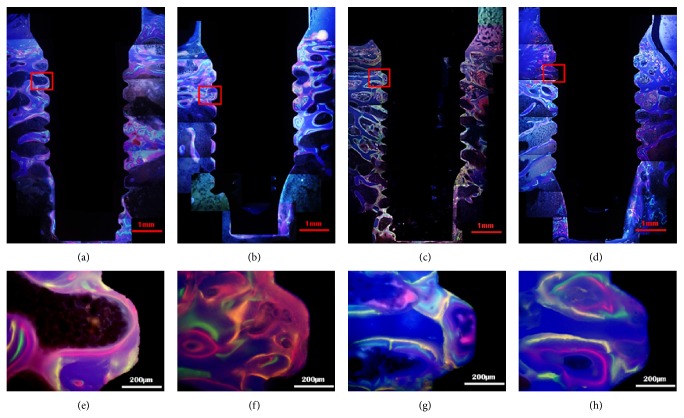
Fluorochrome labeling images under fluorescence microscopy. (a) and (e) control implant at week 4; (b) and (f) nano-CaP implant at week 4; (c) and (g) control implant at week 12; (d) and (h) nano-CaP implant at week 12. Red color is labeled by alizarin red at week 1 or week 3, green color is labeled by calcin green at week 2 or week 6, and yellow color is labeled by oxytetracycline at week 3 or week 9.

Fluorescence microscopy images are shown in Figure 2 and the measurements of bone growth rate are shown in Table 2. Comparing bone growth rates between different time points, the control groups showed that the growth rates at weeks 2 and 3 were marginally significantly higher than that at week 1 (*P* = 0.050), but no significant differences were found at later stages among weeks 3, 6, and 9 (*P* = 0.700). The nano-CaP groups showed a stable bone growth rate in the first 3 weeks (*P* = 0.742), but the growth rates at weeks 6 and 9 were significantly higher than the rate at week 3 (*P* = 0.022).

When compared the nano-CaP and control groups, no significant differences of bone growth rates were found in the first three weeks. At later stages, the bone growth rate of the nano-CaP group was significantly higher than that of control group at week 6 (*P* = 0.021) and week 9 (*P* = 0.001) (Table 3).

### 3.5. Histomorphological Analysis

**Figure 3:**
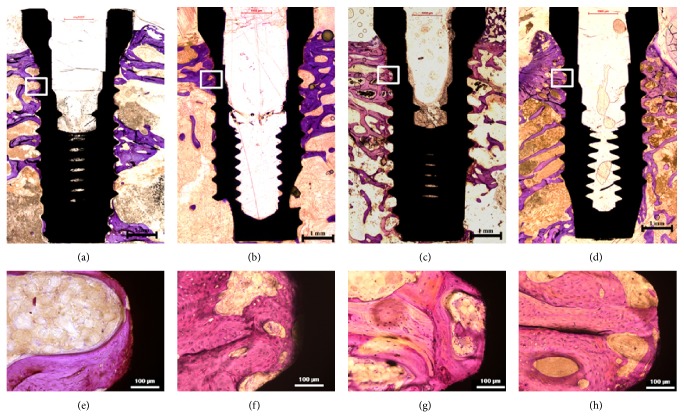
Histological images under light microscopy. (a) and (e) control implant at week 4; (b) and (f) nano-CaP implant at week 4; (c) and (g) control implant at week 12; (d) and (h) nano-CaP implant at week 12.

Histological images showed that implants of control and nano-CaP groups were well integrated with the surrounding bone. No inflammation was observed. The new bone was directly in contact with the implant surface (Figure 3). No change in BIC was found in the control group (*P* = 0.158), while there was a significant increase in BIC in the nano-CaP group from week 4 to week 12 (*P* = 0.009) (Table 2). No significant differences were found between control and nano-CaP groups at week 4 (*P* = 0.390) and at week 12 (*P* = 0.184) (Table 2).

## 4. Discussion

Rabbit has been used in many studies to investigate implant osseointegration in irradiated bone [7, 8, 12]. Our previous study using the same animal model demonstrated a dose-dependent effect of radiation on bone healing around dental implants [8]. The implant stability and bone volume was significantly compromised by a single dose of 15 Gy radiation [8].

Different surface modifications for titanium implants have been advocated to shorten the time of osseointegration [13, 14]. Calcium phosphate (CaP) is reported to promote cell attachment, proliferation, differentiation, and the production of extracellular matrix (ECM) in vitro [15, 16]. The favorable property of CaP coating might be due to the similarity of chemical composition between CaP coating and natural bone [17]. CaP coatings on titanium surface simulate the organic and inorganic components of natural bone tissue, which guides bone formation along the implant-bone interface [18]. CaP dissolved and delivered into the peri-implant region also raises the saturation level of body fluid and results in deposition of biological apatite on the surface of implants [19]. Nano-CaP implants, on the other hand, might have the potential of enhancing the “secondary” stability. This might be clinically useful not only for patients who had radiotherapy but also in other compromised bone conditions such as osteoporosis or inadequate bone height.

A number of studies have investigated discrete crystalline deposition (DCD) of calcium phosphate on implant surface; however, its effect on osseointegration was controversial. Most of the studies showed that nano-CaP coating of titanium surface could promote bone formation on implant surface, raise the torque required to remove implants, and increase BIC [20–23]. However, some studies found that the nano-CaP coating did not enhance early bone tissue integration in animal [24, 25] and clinical studies [26]. The discrepancy might be due to the different experimental model and time points for assessment. While most of the studies which assessed the osseointegration at or before week 4 did not find significant difference between nano-CaP group and control group, the long-term studies with the observation done after several months detected a difference. The present study showed no difference in secondary stability value at week 4, while the significantly higher ISQss value was detected in nano-CaP group at week 12. Our study also found that the bone growth rate was significantly higher in nano-CaP group at week 6 and week 9. The identical results of increased ISQ and bone growth rate at the late stage of the present study suggested that nano-CaP surface modification may improve osseointegration in longer term rather than in early stage after implant placement.

The present study did not find any differences in BIC and BV/TV between nano-CaP and control groups. This might be due to the limitation of sample size and observation time. A further study using a larger sample size with longer observation period is necessary.

## 5. Conclusions

Titanium implant surface modified with CaP nanocrystals may have potential to improve implant osseointegration in irradiation compromised bone. Further study with larger sample size and longer observation period is necessary.

## Figures and Tables

**Table 1:** Timetable of radiation, implant surgery, injection, and sacrifice on the rabbits in different groups.

	Rabbit no.	Radiation	Implant surgery and measure ISQ	Inject alizarin red	Inject calcin green	Inject oxytetracycline	Sacrifice, measure ISQ, and fixation
Control group	4	Week 1	Week 0	Week 1	Week 2	Week 3	Week 4
Nano-CaP group	4						

Control group	4	Week 1	Week 0	Week 3	Week 6	Week 9	Week 12
Nano-CaP group	4						

**Table 2:** Values of implant primary stability (ISQps), secondary stability (ISQss), ratio of bone volume to total volume (BV/TV), and percentage of bone to implant contact (BIC).

	ISQps	ISQss	BV/TV (%)	BIC (%)
Control group (4 w)	65.25 ± 8.01	71.25 ± 4.98	55.57 ± 8.08	61.8 ± 8.1
Nano-CaP group (4 w)	63.75 ± 6.23	69.63 ± 5.15	53.31 ± 7.35	57.9 ± 8.8
Control group (12 w)	63.13 ± 5.54	74.25 ± 6.14	64.16 ± 8.20	64.3 ± 9.7
Nano-CaP group (12 w)	64.38 ± 7.37	78.25 ± 8.63	65.59 ± 8.54	70.2 ± 8.6

**Table 3:** The mean and SD of bone growth rate (*µ*m/day).

Group	Week 1	Week 2	Week 3
Control group (4 w)	1.21 ± 0.54	1.81 ± 0.47	1.58 ± 0.39
Nano-CaP group A (4 w)	1.24 ± 0.30	1.37 ± 0.54	1.20 ± 0.51

	Week 3	Week 6	Week 9

Control group (12 w)	1.07 ± 0.27	1.22 ± 0.42	1.19 ± 0.41
Nano-CaP group (12 w)	1.33 ± 0.53	2.74 ± 1.60	2.85 ± 0.97
